# Low Expression of Sirtuin 1 in the Dairy Cows with Mild Fatty Liver Alters Hepatic Lipid Metabolism

**DOI:** 10.3390/ani10040560

**Published:** 2020-03-27

**Authors:** Yu Li, Suping Zou, Hongyan Ding, Ning Hao, Yingying Huang, Jishun Tang, Jianbo Cheng, Shibin Feng, Jinchun Li, Xichun Wang, Jinjie Wu

**Affiliations:** 1 College of Animal Science and Technology, Anhui Agricultural University, Hefei 230036, China; lydhy2014@ahau.edu.cn (Y.L.); 18855952196@163.com (S.Z.); dinghy1988@163.com (H.D.); hao621065@163.com (N.H.); huangyingying@ahau.edu.cn (Y.H.); chengjianbofcy@163.com (J.C.); luyifsb@163.com (S.F.); jinchunli64@163.com (J.L.); 2 Anhui Province Key Laboratory of Veterinary Pathobiology and Disease Control, Hefei 230036, China; 3 Institute of Animal Husbandary and Veterinary Medicine, Anhui Academy of Agriculture Sciences, Hefei 230031, China; tjs157@163.com

**Keywords:** dairy cow, fatty liver, lipid metabolism, oxidative stress, SIRT1

## Abstract

**Simple Summary:**

Sirtuin 1 (SIRT1), a NAD-dependent histone deacetylase, is involved in oxidative stress and lipid metabolism regulation. Limited studies exist regarding the role of SIRT1 in lipid metabolism disorder in periparturient dairy cows. This study explores the effect of hepatic steatosis on the expression of the SIRT1 gene and protein and the proteins encoded by the genes downstream to it, all of which are involved in lipid metabolism in the liver. Control cows (n = 6, parity 3.0 ± 2.0, milk production 28 ± 47 kg/d) and mild fatty liver cows (n = 6, parity 2.3 ± 1.5, milk production 20 ± 6 kg/d) were retrospectively selected based on liver triglycerides (TG) content (% wet liver). The present study indicates that low SIRT1 expression caused by hepatic steatosis promotes hepatic fatty acid synthesis and inhibits fatty acid β-oxidation. We believe that our study makes a significant contribution to the literature because it demonstrates that hepatic steatosis is associated with increased hepatic fatty acid synthesis, inhibited fatty acid β-oxidation and reduced lipid transport.

**Abstract:**

Dairy cows usually experience negative energy balance coupled with an increased incidence of fatty liver during the periparturient period. The purpose of this study was to investigate the effect of hepatic steatosis on the expression of the sirtuin 1 (SIRT1), along with the target mRNA and protein expressions and activities related to lipid metabolism in liver tissue. Control cows (n = 6, parity 3.0 ± 2.0, milk production 28 ± 7 kg/d) and mild fatty liver cows (n = 6, parity 2.3 ± 1.5, milk production 20 ± 6 kg/d) were retrospectively selected based on liver triglycerides (TG) content (% wet liver). Compared with the control group, fatty liver cows had greater concentrations of cholesterol and TG along with the typically vacuolated appearance and greater lipid droplets in the liver. Furthermore, fatty liver cows had greater mRNA and protein abundance related to hepatic lipid synthesis proteins sterol regulatory element binding proteins (SREBP-1c), long-chain acyl-CoA synthetase (ACSL), acyl-CoA carbrolase (ACC) and fatty acid synthase (FAS) and lipid transport proteins Liver fatty acid binding protein (L-FABP), apolipoprotein E (ApoE), low density lipoprotein receptor (LDLR) and microsomal TG transfer protein (MTTP) (*p* < 0.05). However, they had lower mRNA and protein abundance associated with fatty acid β-oxidation proteins SIRT1, peroxisome proliferator-activated receptor co-activator-1 (PGC-1α), peroxisome proliferator–activated receptor-α (PPARα), retinoid X receptor (RXRα), acyl-CoA 1 (ACO), carnitine palmitoyltransferase 1 (CPT1), carnitine palmitoyltransferase 2 (CPT2) and long- and medium-chain 3-hydroxyacyl-CoA dehydrogenases (LCAD) (*p* < 0.05). Additionally, mRNA abundance and enzyme activity of enzymes copper/zinc superoxide dismutase (Cu/Zn SOD), catalase (CAT), glutathione peroxidase (GSH-Px) and manganese superoxide dismutase (Mn SOD) decreased and mRNA and protein abundance of p45 nuclear factor-erythroid 2 (p45 NF-E2)-related factor 1 (Nrf1), mitochondrial transcription factor A (TFAM) decreased (*p* < 0.05). Lower enzyme activities of SIRT1, PGC-1α, Cu/Zn SOD, CAT, GSH-Px, SREBP-1c and Mn SOD (*p* < 0.05) and concentration of reactive oxygen species (ROS) were observed in dairy cows with fatty liver. These results demonstrate that decreased SIRT1 associated with hepatic steatosis promotes hepatic fatty acid synthesis and inhibits fatty acid β-oxidation. Hence, SIRT1 may represent a novel therapeutic target for the treatment of the fatty liver disease in dairy cows.

## 1. Introduction

Dairy cows undergo negative energy balance (NEB) when they transition from late gestation to early lactation, during which ketosis, fatty liver and metritis are likely to occur [1,2]. The occurrence of fatty liver in dairy cows increases treatment costs and culling and decreased milk production [3]. In addition, fatty liver disease develops when increased infectious and metabolic diseases are likely to occur [4,5]. For these reasons, fatty liver has become a major international health burden in dairy cows. 

The protein sirtuin 1 (SIRT1), an NAD^+^-dependent deacetylase, and deacetylates histones including transcription factors lead to the regulation of metabolism, oxidative stress and cellular survival [6]. SIRT1 is highly sensitive to intracellular redox status and provides cells with the ability to tolerate oxidative stress. SIRT1 protects cells from oxidative stress by increasing the activity of catalase [7,8,9]. Oxidative stress was increased by SIRT1 inhibitor (Ex 527) treatment and decreases in SIRT1 expression were observed in neurons [10]. Ex 527 attenuated the activity of histone deacetylase (HDAC) and increased the degree of myocardial injury during oxidative stress [11]. Accumulating evidence suggests that SIRT1 plays a protective role in the process of oxidative stress and that oxidative stress was induced by excessive fat mobilization in perinatal cows. We observed in our studies that oxidative stress was induced by high non-esterified fatty acid (NEFA) concentrations in dairy cows [12,13]. To date, the effect of SIRT1 on the oxidative stress in dairy cows remains unclear.

It was reported that SIRT1 plays a critical role in lipid metabolism by modulating the activity of transcription factors [14]. In mice and obese patients fed with a high-fat diet, the activity and expression of SIRT1 were significantly decreased, which attenuated the mobilization of fatty acids and promoted the occurrence of metabolic disorders [15,16,17]. In addition, mounting evidence indicates that a long-term, high-calorie-diet induced liver steatosis is promoted by increasing the expression of SIRT1 in animals [18,19]. Here, we report that the knocked down expression or overexpression of SIRT1 results in changes in the lipid/cholesterol (Chol) levels in the serum and liver, and causes accumulation of lipids in the liver, a process leading to hepatic steatosis [20,21]. Evidence is emerging that SIRT1 plays a vital role in lipid metabolism in the liver. However, the underlying molecular mechanisms, the effect of hepatic steatosis on the expression of SIRT1 genes and proteins and the expressions and activities of downstream lipid-metabolism-related proteins and key enzymes, have not yet been fully clarified. 

Therefore, we aimed to investigate the change of expression levels of SIRT1 and downstream lipid-metabolism-related proteins in the pathogenesis of fatty liver disease in dairy cows, thereby providing a theoretical and experimental basis for revealing the pathogenesis of fatty liver in dairy cows and searching for therapeutic targets. 

## 2. Materials and Methods 

### 2.1. Ethics Statement

This study was conducted following the recommendations in the Guide for Care and Use of Laboratory Animals of the National Institutes of Health. All experimental procedures were approved by the Institutional Animal Care and Use Committee of Anhui Agricultural University (permit number: 20170624). All surgeries were performed under anesthesia and all efforts were made to minimize suffering. Briefly, cows were anesthetized with thiamylal sodium along the midline skin of the abdomen. Then the abdominal cavity was opened, the liver was obtained and placed on a sterile bench treated with sterile 0.9% sodium chloride solution for removing bloodstains on the surface.

### 2.2. Animals

Lactating Holstein multiparous cows with the same breed, age and having similar milk production characteristics and body condition scores were selected from a commercial dairy farm located in Hefei city, Anhui province, China. The rectal temperature, respiratory rate and pulse rate for each cow was performed by a skilled veterinarian to ensure the cows had no other co-morbidities. Clinical observations and other disease conditions were recorded (Table 1). The cows were fed ad libitum with a total mixed ration (TMR) forage that met the animals’ nutrient requirements (Table 2) and had free access to get water. A total of 34 dairy cows were screened by liver biopsy using a liver puncture needle based on the hepatic TG content (% wet liver, <1% was considered to be healthy cows, 1–10% was considered to be mild fatty liver cows), which is the standard for fatty liver diagnosis [22,23]. Equal numbers of control (n = 6) and fatty liver cows and were retrospectively selected based on liver triglycerides (TG) content. Finally, 12 dairy cows were slaughtered for collecting liver tissue samples and processed as part of the normal work of a commercial abattoir (Hefei, China).

### 2.3. Average Milk Production Collection and Milk Components Analysis

As described in the previous study, the daily dry matter intake (DMI) for individual cows was calculated by subtracting the orts from the feed offered [24]. Cows were milked at 06:00, 14:00 and 20:00 and milk production was recorded at each milking. At d 70 in milk, the liver was collected. The milk aliquots were stored at 4 °C until analysis. Milk components include fat, protein, lactose, total solids and milk urea nitrogen (MUN) concentrations were determined by mid-infrared spectrophotometry method on a MilkoTMScan (MilkoScan Type FT120, Foss Electric, Hillerød, Denmark). Somatic cell counts (SCC) were conducted on a Fossomatics 5000 (Foss Analytical A/S; Foss Electric, Hillerød, Denmark). Both 4% fat corrected milk (FCM) and energy corrected milk (ECM) were calculated and the equations were as follows: 4% FCM = 0.4 × milk (kg) + 15 × fat (kg) and ECM = 0.327 × milk (kg) + 12.95 × fat (kg) + 7.20 × protein (kg). Feed efficiency was calculated as the daily milk yield/kg of feed DMI on an individual cow basis.

### 2.4. Liver chemistry measurement

The liver Chol and TG measurements were performed as previously described [25]. In brief, frozen liver tissue (50 mg) was homogenized with 1 mL lysis buffer. The Chol contents were measured with Chol assay kit, while TG contents were determined using TG kits (IDEXX Vet Tests, Westbrook, ME, USA). Total protein concentration was measured using bicinchoninic acid (BCA) method. 

### 2.5. ELISA Assay for the Contents of SIRT1 and Downstream Lipid- metabolism-related Proteins and Key Enzymes

Liver samples were homogenized in ice-cold lysis buffer (0.05 M-phosphosaline, pH 7.4, containing 0.025 M EDTA, 0.08% sodium azide and 0.05% Triton X-100) supplemented with protease inhibitor cocktail (P1860, Sigma-Aldrich Co., St. Louis, MO, USA). Homogenates were incubated for 10 min at 4 °C and centrifuged at 14,000 × g at 4 °C for 15 min. The supernatants were separated into two aliquots (500 μL each). One was immediately used for analysis, while the other was stored at −80 °C until further analysis. SIRT1, SREBP-1c, PGC-1α and the concentrations of CAT, Cu/Zn-SOD, Mn-SOD, GSH-Px, GSH, GSSG and ROS (oxidation and antioxidation key indexes) levels in liver supernatants were determined by ELISA provided by Shanghai Bluegene Biotech Co., Ltd. according to the manufacture’s protocol. Total protein concentration was measured using the bicinchoninic acid (BCA) method. 

### 2.6. Liver Histology

Tissue samples obtained from biopsy or necropsy were fixed in 10% formalin and embedded in paraffin. Sections were prepared with a thickness of 2–3 μm, stained with hematoxylin/eosin (H&E), and examined. Accumulation of triglyceride (TG) content in the liver was visualized by Oil Red O (Sigma-Aldrich) staining. Slides were viewed and images were taken using an Olympus BX41TF System Microscope (Olympus Corporation, Tokyo, Japan). 

### 2.7. Total RNA Isolation and qRT-PCR

The liver was homogenized in TRIzol reagent (Invitrogen Life Technologies, Grand Island, NY) and total RNA was isolated according to the manufacturer’s instructions. The RNA concentration and quality were measured by K5500 Micro-Spectrophotometer (Kaiao, Beijing, China). RNA was reverse-transcribed into cDNA according to the Reverse Transcription Systems instructions (TaKaRa, Dalian, China). According to the GenBank sequence, the primer sequences of the target genes were designed using the software Primer Premier 5.0 and β-actin was used as a reference gene (Table 3). The amplification products were analyzed by 1.5% agarose gel electrophoresis and a gel imaging and analysis system (UVItec, Cambridge, UK). The mRNA expression levels were determined by quantitative reverse-transcription polymerase chain reaction (qPCR) via an ABI prism 7500 Real-Time PCR system (Applied Biosystems). The mRNA relative abundance was calculated according to the method of Pfaffl and was normalized to the mean expression of β-actin and results (fold changes) were expressed as 2^−ΔΔCt^ [26]. 

### 2.8. Western Blot Analysis

Liver tissue homogenates were solubilized in SDS sample buffer. As previously described, liver total protein was extracted by a commercial kit (Sangon Biotech, China) [12]. Protein concentrations were measured by BCA method. A total of 50 μg protein for each sample was separated by SDS-PAGE. The required gels were cut and transferred into a PVDF membrane (Shanghai Jinsheng Biological Engineering Co, Shanghai, China) with electrophoresis buffer. By using appropriate antibodies (Abs), immunoreactive bands were visualized by a gel imaging system (Bio-Rad, Hercules, CA, USA). Primary antibodies against ACO (YN0401), FAS (YM1224), ACCα (YT0074), p-ACCα (YP0620), ACSL1 (YN0827), LDLR (YN2236), RXRα (YN0018), Nrf1 (YT3188) and TFAM (YT2916) were purchased from ImmunoWay Biotechnology Company (Newark, DE, USA). Primary antibodies against CPT1 (15184-1-AP) and CPT2 (26555-1-AP) were purchased from Proteintech Group (Chicago, IL, USA). Primary antibodies against PPARα (SC-1985), SREBP-1c (SC-365513) and ApoE (SC-31822) were purchased from Santa Cruz Biotechnology (Santa Cruz, CA, USA). Primary antibody against SIRT1 (D1D7) was purchased from Cell Signaling Technology (Danvers, MA, USA). Antibodies against PGC-1α (ab54481) was purchased from Abcam (Cambridge, MA, USA). Horseradish peroxidase-conjugated secondary antibodies were purchased from Wuhan Boster Biological Engineering Co. (Wuhan, China). The results were analyzed using Quantity One software (Bio-Rad, Hercules, CA, USA). The protein levels were normalized by blots against β-actin.

### 2.9. Statistical Analysis 

Data are presented as mean ± SEM. Differences between the mean values of the normally distributed and homogeneity of variance data were analyzed using a two-tailed Student’s t-test. The results are exploratory and the differences were considered significant at *p* < 0.05 or *p* < 0.01 in all the studies.

## 3. Results

### 3.1. Milk Production, Milk Component and SCC

As shown in Table 4, the DMI and milk yield in the fatty liver group were significantly lower than those in the control group (*p* < 0.01). The milk protein and MUN were significantly reduced (*p* < 0.01) and SCCs were significantly increased (*p* < 0.01) in the fatty liver group.

### 3.2. Chol and TG Concentrations in the Control and Fatty Liver Dairy Cows

**Figure 1:**
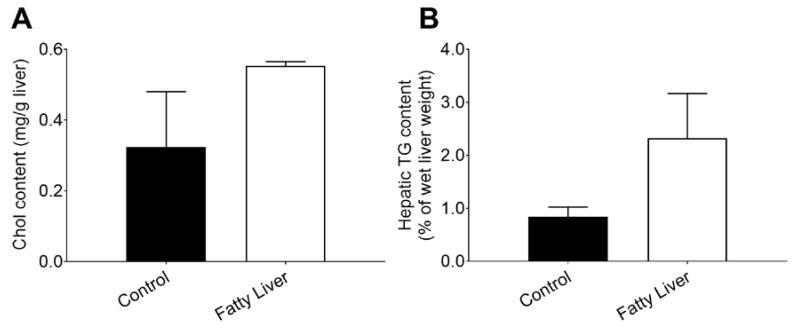
Cows with fatty livers exhibit over the induction of hepatic lipid synthesis (**A**,**B**). Dairy cows were classified according to the hepatic triglyceride (TG) content into a control group (n = 6) and a fatty liver group (n = 6) dairy cows (B); The data presented are the mean ± SEM. * *p* < 0.05; ** *p* < 0.01.

Compared with the control group, the concentrations of Chol (Figure 1A) and TG (Figure 1B) were increased in the fatty liver group. 

### 3.3. Histological Analysis of Liver

**Figure 2:**
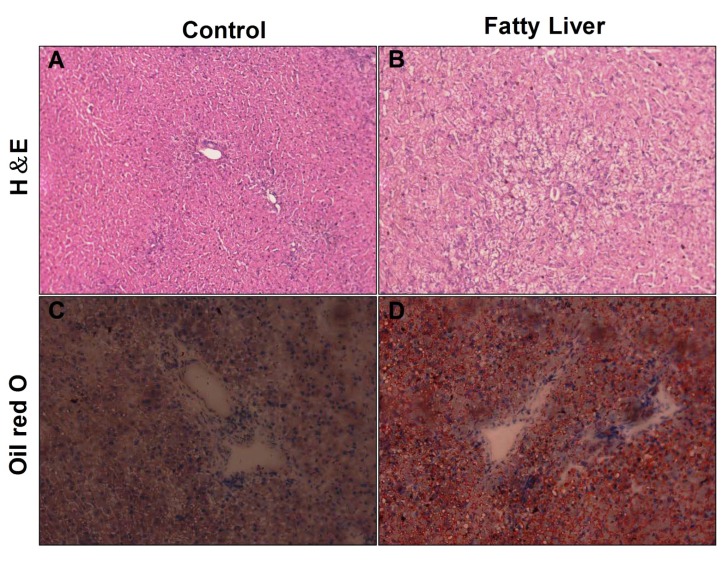
Assessment of hepatic histology in the liver of the control group and fatty liver group. (**A**,**B**) Hematoxylin and Eosin staining of liver sections from the control group and fatty liver group. Original magnification: 20× (**C**,**D**) Liver sections were stained with Oil Red O and Hematoxylin stain for nuclei. Original magnification: 20×.

Isolated cow livers were subjected to H&E staining to evaluate their hepatic steatosis status. It was characterized by a typically vacuolated appearance of the lipid-laden hepatocytes in the H&E-stained sections of the liver (Figure 2A,B) and confirmed by the demonstration of more intrahepatic lipid on oil red O staining of the frozen sections (Figure 2C,D). Steatosis was primarily centrilobular and either microvesicular or mixed microvesicular/ macrovesicular. In fatty liver cows, the oil red O stained lipid droplets occupied an area larger and more than that which was in the control group cows. These results suggest that cows with fatty liver have a hepatic injury. 

### 3.4. Effects of Hepatic Steatosis on Enzyme Activities of SIRT1, SREBP-1c, PGC-1α and Redox Index 

**Figure 3:**
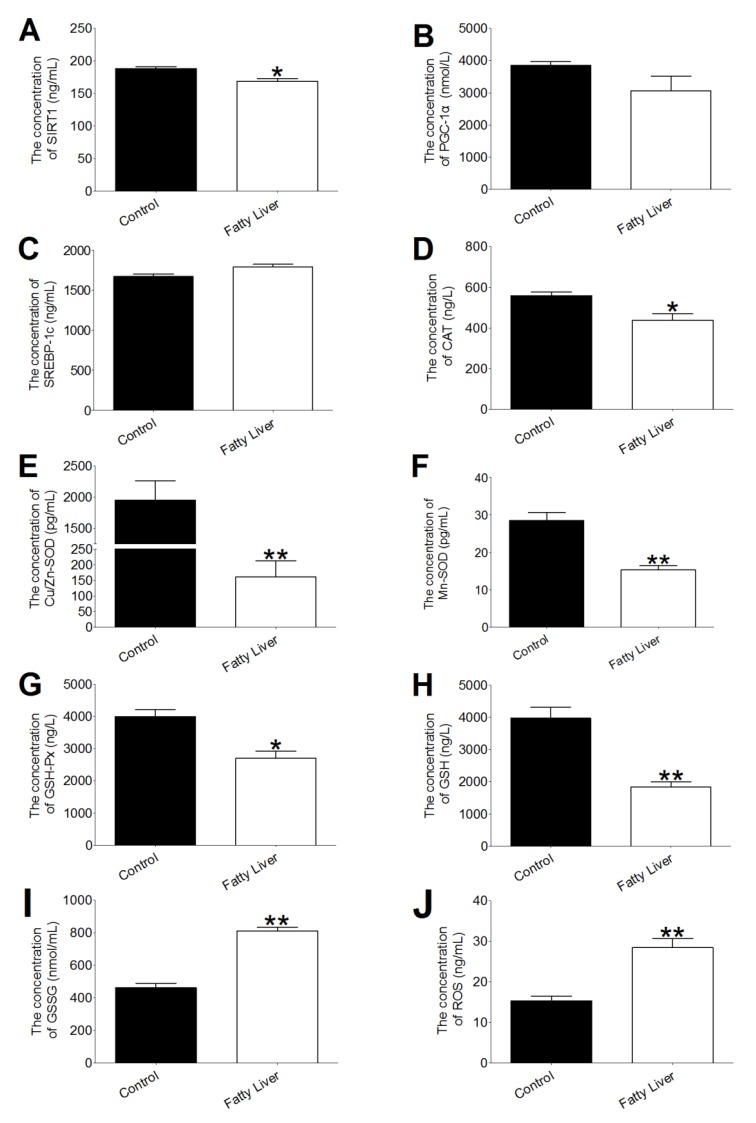
Effect of hepatic steatosis on the activities of hepatic sirtuin 1 (SIRT1), sterol regulatory element binding proteins (SREBP-1c), peroxisome proliferator-activated receptor γ coactivator-1 alpha (PGC-1α), catalase (CAT), copper-and zinc-containing superoxide dismutase (Cu/Zn SOD), manganese superoxide dismutase (Mn-SOD), glutathione peroxidase (GSH-Px) and the concentrations of glutathione (GSH), reduce glutathione disulfide (GSSG) and reactive oxygen species (ROS). Effect of lipid deposition on SIRT1, SREBP-1c and PGC-1α concentrations in control (n = 6) and fatty liver (n = 6) group dairy cows (**A**–**C**); Effect of lipid deposition on antioxidant index concentrations in control (n = 6) and fatty liver (n = 6) group dairy cows (**D**–**H**); Effect of lipid deposition on oxidative indexes concentrations in dairy cows (**H**–**J**). The data presented are the mean ± SEM. * *p* < 0.05; ** *p* < 0.01.

The effects of hepatic steatosis on hepatic SIRT1, sterol regulatory element binding transcription factor 1 (SREBP-1c), peroxisome proliferator-activated receptor γ coactivator-1 alpha (PGC-1α), oxidation and antioxidation activities in the liver are shown in Figure 3. The SIRT1, catalase (CAT) and glutathione peroxidase (GSH-Px) activities of the fatty liver group were significantly reduced (*p* < 0.05). The enzyme activities of Cu/Zn superoxide dismutase (SOD), Mn SOD and the content of glutathione (GSH) in the fatty liver group were significantly lower than those in the control group (*p* < 0.01). The GSSG (Figure 3I) contents in the fatty liver were higher than those in the control group liver (*p* < 0.01). The cow livers in the fatty liver group generated more reactive oxygen species (ROS) compared to the cow livers in the control group (*p* < 0.01, Figure 3J). Besides, mRNA abundance of CAT, GSH-Px and Mn SOD have a greater decrease (*p* < 0.01, Table 5) compared with the fatty liver group.

### 3.5. Effect of Hepatic Steatosis on the Protein and mRNA Abundance of Fatty Acid Oxidation Proteins

**Figure 4:**
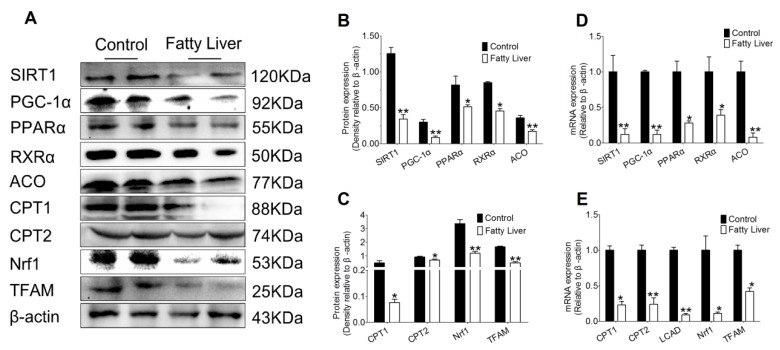
Lipid deposition hinders the fatty acid oxidation pathway in cow liver. (**A**–**C**) Levels of the protein levels of SIRT1, PGC-1α, peroxisome proliferator-activated receptor alpha (PPARα), retinoid X receptor alpha (RXRα), acyl coenzyme A oxidase (ACO), carnitine palmitoyltransferase 1 (CPT1), carnitine palmitoyltransferase 2 (CPT2), nuclear respiratory factor 1 (Nrf1) and transcription factor A (TFAM) in control (n = 6) and fatty liver (n = 6) group dairy cows were determined by western blotting. (**D**,**E**) Total RNA was extracted from liver samples and the expressions of genes involved in the fatty acid oxidation pathway in control (n = 6) and fatty liver (n = 6) group dairy cows were determined by real-time RT-PCR. The data presented are the mean ± SEM. * *p* < 0.05; ** *p* < 0.01.

To explore the effect of hepatic steatosis on hepatic lipid metabolism, we measured the protein and mRNA abundance of different enzymes involved in lipid acid oxidation process. As shown in Figure 4A–C, compared with the control group, a greater downregulation of SIRT1 took place in liver tissues of the fatty liver group (*p* < 0.01). Additionally, hepatic steatosis (fatty liver group) led to a lower protein abundance of SIRT1 related transcription factors PGC-1α and peroxisome proliferator-activated receptor alpha (PPARα) (*p* < 0.05 or *p* < 0.01). Subsequently, the transcription factors target genes retinoid X receptor alpha (RXRα), acyl coenzyme A oxidase (ACO), carnitine palmitoyltransferase 1 (CPT1), carnitine palmitoyltransferase 2 (CPT2), nuclear respiratory factor 1 (Nrf1) and transcription factor A (TFAM) decreased in the fatty liver group (*p* < 0.05 or *p* < 0.01). 

Meanwhile, mRNA abundance of fatty acid oxidation proteins, including SIRT1, PGC-1α, PPARα, RXRα, ACO, CPT1, CPT2, long chain acyl-CoA dehydrogenase (LCAD), Nrf1 and TFAM were observed. As shown in Figure 4D, mRNA abundance of SIRT1 and its downstream transcription factors PGC-1αand PPARα decreased in the liver tissue of the fatty liver group compared with the control group (*p* < 0.05 or *p* < 0.01). Furthermore, compared with the control group, hepatic steatosis (fatty liver group) led to decreased mRNA abundance of RXRα, ACO, CPT1, CPT2, LCAD, Nrf1 and TFAM (*p* < 0.05 or *p* < 0.01). 

### 3.6. Hepatic Steatosis Enhances Protein and mRNA Abundance of Hepatic Lipogenesis

**Figure 5:**
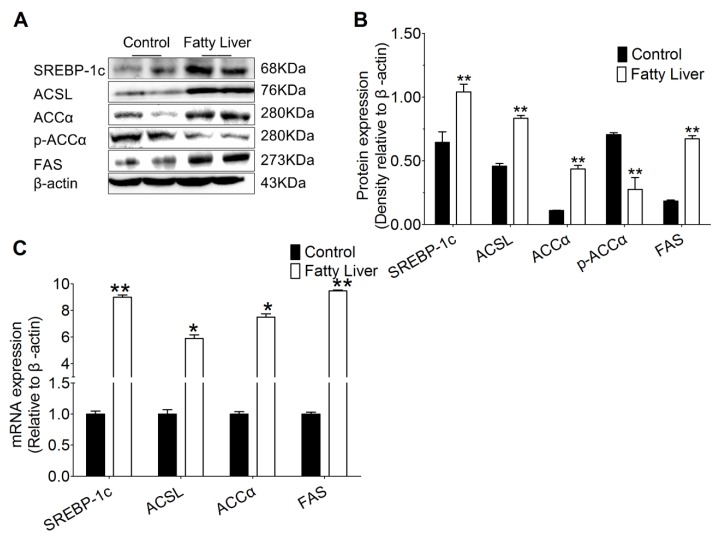
Lipid deposition enhances the hepatic lipogenesis pathway in the cow liver. (**A**,**B**) Protein levels of SREBP-1c, acyl-CoA synthetase long chain family member 1 (ACSL1), acetyl-CoA carboxylase alpha (ACCα), p-ACCα and fatty acid synthetase (FAS) in control (n = 6) and fatty liver (n = 6) group dairy cows were determined by western blotting. (**C**) Total RNA was extracted from liver samples and the expressions of the genes involved in free fatty acid (FFA) and triglycerides (TG) biosynthesis in control (n = 6) and fatty liver (n = 6) group dairy cows were determined by real-time RT-PCR. The data presented are the mean ± SEM. * *p* < 0.05; ** *p* < 0.01.

As shown in Figure 5A,B, compared with the control group, hepatic steatosis (fatty liver group) increased protein abundance of SIRT1 related transcription factor SREBP-1c (*p* < 0.01). Moreover, the transcription factors target genes including acyl-CoA synthetase long chain family member 1 (ACSL1) and fatty acid synthetase (FAS) increased in liver tissue of fatty liver group (*p* < 0.01). It is noteworthy that, protein abundance of acetyl-CoA carboxylase alpha (ACCα) being greater increase compared with the control group, hepatic steatosis (fatty liver group) led to lower p-ACCα and p-ACCα/ACCα. 

Compared with the control group, mRNA abundance of SREBP-1c and its target genes ACSL and FAS had a greater increase in liver tissue of the fatty liver group (*p* < 0.01, Figure 5C). Hepatic steatosis (fatty liver group) also led to increased mRNA abundance of ACCα compared with control. 

### 3.7. Effect of Hepatic Steatosis on Hepatic Lipid Transport Related Proteins

**Figure 6:**
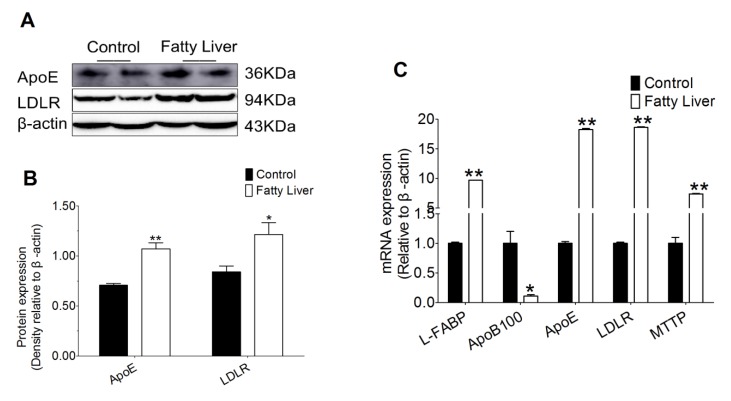
Lipid deposition promotes the lipid transport pathway in the cow liver. (**A**,**B**) Levels of the proteins apolipoprotein E (ApoE) and low density lipoprotein receptor (LDLR) in control (n = 6) and fatty liver (n = 6) group dairy cows were determined by western blotting. (**C**) Total RNA was extracted from liver samples and the expressions of the genes involved in the lipid transport pathway in control (n = 6) and fatty liver (n = 6) group dairy cows were determined by real-time RT-PCR. The data presented are the mean ± SEM. * *p* < 0.05; ** *p* < 0.01.

We also found that the protein abundance of hepatic lipid transport including apolipoprotein E (ApoE) and low density lipoprotein receptor (LDLR) in the fatty liver group were increased, compared with the control group (*p* < 0.05 or *p* < 0.01, Figure 6A,B). Liver tissue in the fatty liver group had greater mRNA abundance of liver fatty acid binding proteins (L-FABP), apolipoprotein E (ApoE), low density lipoprotein receptor (LDLR) and microsomal triglyceride transfer protein (MTTP) compared with the control group (*p* < 0.01, Figure 6C). However, mRNA abundance of apolipoprotein B 100 (ApoB100) decreased in the liver tissue of the fatty liver group.

## 4. Discussion

The present study provides the basis for further animal and clinical studies to validate the mechanism, namely SIRT1, as a nicotinamide adenine dinucleotide (NAD^+^, NADH)-dependent class III protein deacetylase, which is a key regulator of energy homeostasis in response to nutrient availability. Negative energy balance is a normal incidence in dairy cows during the transition from late gestation to early lactation [27]. It occurs because the energy demands for early milk production cannot be completely met by feed intake [3], during which most infectious and metabolic diseases are likely to occur, such as hepatic steatosis [1,4]. Our study showed that fatty liver significantly decreased DMI, milk yield, the milk protein and MUN, and significantly increased SCC, which indicates that fatty liver decreased the milk production and negatively affect milk quality. Furthermore, the protein, as well as the levels of Chol and TG were higher in the livers of fatty liver dairy cows. The liver is of immediate importance in maintaining health and plays a key role in the development of impaired metabolic regulation [28]. Previous studies have highlighted the importance of SIRT1 in cell metabolism, but mainly focused on the upstream regulation of SIRT1, and did not explore the mechanisms regarding SIRT1, lipid metabolism and oxidative stress [29,30,31]. In the present study, our results showed that hepatic steatosis decreased SIRT1 activity that facilitated hepatic fatty acid synthesis and inhibited fatty acid oxidation and lipid transport. 

While SIRT1 is mainly localized in the nucleus, it is also present in the cytosol [30]. It deacetylates a variety of protein targets [32]. Hepatic SIRT1 deficiency in mice impairs lipid metabolism and results in hepatic steatosis [31,33]. Treatment with resveratrol, a SIRT1 activator, ameliorates fatty liver with a reduction in the expression of lipogenic enzymes in mice exhibiting obesity and insulin resistance [34,35,36]. Numerous studies have demonstrated that SIRT1 can act as a signaling molecule that promotes lipid metabolism by affecting the expressions of the genes controlling fatty acid synthesis, fatty acid oxidation and lipid transport [37]. It was reported that fatty livers facilitate the development of hepatic steatosis by upregulating primarily the lipogenic pathway genes via the SREBP-1c signaling pathway and down-regulating the lipid oxidizing genes predominantly via the SIRT1 and PGC-1α signaling pathways, which is in agreement with our results. Concomitant with the occurrence of hepatic steatosis, oxidative stress is an established risk factor for the development of lipid metabolism disorders at least partly through the SIRT1-SREBP-1c/ PGC-1α signaling pathway.

Previous studies suggest that excess lipid accumulation is associated with fatty liver through oxidative stress [38,39]. Also, it is found that oxidative stress participates in liver disease both in humans and animal models [40,41]. Oxidative stress is caused by an imbalance between the generation of ROS and the defense capabilities of antioxidants [42]. Previous studies on dairy cows have also demonstrated that elevated NEFA during the periparturient period largely activates oxidative stress [43]. Further results from in vivo study indicate that the primary approaches to prevent the occurrence of fatty liver disease in cows are counteracted oxidative damage [44]. However, most studies focus on the blood oxidative and antioxidative levels to assess the oxidative stress status of dairy cows. Fortunately, the oxidative status of ketotic dairy cows had been evaluated by liver biopsies [12]. As shown in the present study, the activities of hepatic CAT and GSH-Px and the GSH content had a greater reduction in dairy cows with fatty livers. However, the hepatic GSSG and ROS contents were significantly increased in dairy cows with fatty livers compared with the control group. Herein, our results indicated that dairy cows with fatty livers showed redox imbalance, which was in agreement with a previous report. 

Fatty liver disease mainly occurs when fat accumulation is caused by impairing fatty acid oxidation along with increased lipid synthesis [44]. PPARαregulates the lipid oxidation genes expression involved in lipid oxidation, including ACO, CTP1 and CPT2 [45,46]. ACO has been considered as the rate-limiting enzyme for fatty acid oxidation [47]. CTP1 and CPT2 are thought to be the key enzymes in the process of transferring fatty acids into the mitochondria for β-oxidation [45]. In bovine hepatocytes treated 0.15, 0.30 and 0.45 mM non-esterified fatty acids (NEFA) induced the increased PPARα, ACO, CPT1 and CPT2 relative mRNA expression [48]. However, in another vivo study, high-fat diet induced the decreased expression of ACO and CPT2, but had a slight decrease in the CPT2 expression in mice [49]. In the present study, we demonstrated that fatty liver led to a greater reduction of PPARα, ACO, CPT1 and CPT2 protein and mRNA abundance. Our data indicate that hepatic steatosis impaired fatty acid oxidation. PGC-1α plays a crucial role in energy metabolism regulation and stimulates mitochondrial biogenesis via regulating NRF1 and TFAM [50,51]. RXRα was recognized as the receptor of PGC-1α [52]. LCAD, a mitochondrial enzyme, is involved in the branched chain and unsaturated fatty acids oxidation. In this study, the protein and mRNA abundance of hepatic PGC-1α, NRF1, TFAM and RXRα was decreased in fatty liver dairy cows compared with the control group. However, it was reported that the protein levels of PGC-1α decreased in mice with fatty liver followed by reduction of NRF1 and TFAM, the mRNA abundance of PGC-1α increased [53]. The discrepancies between the previous study and our results attribute to different animal species. The mRNA abundance of LCAD had a greater reduction in the dairy cows with fatty livers. These results show that it is highly likely that PGC-1α, NRF1, TFAM and LCAD are associated with fatty liver disease in dairy cows. 

SREBP-1c is considered as an important activator of lipid synthesis by regulating lipogenic genes ACSL, ACCα and FAS [54]. ACSL is a key enzyme and involved in the first step of fatty acid metabolism [55]. ACCα is thought to synthesis long chain fatty acids [56]. FAS, a key lipogenic enzyme, catalyzes the long chain fatty acid synthesis [57]. Moreover, the protein and mRNA abundance of SREBP-1c and its downstream gene ACSL, ACCα and FAS were significantly increased, but the phosphorylation of ACCα decreased. In the present study, the mRNA abundance of L-FABP, a lipid transport protein, markedly increased in the dairy cows with fatty liver. ApoE, ApoB100 and MTT are used to the lipid synthesis and VLDL assembly [58]. Otherwise, ApoE and ApoB100 play a key role in cholesterol transport by binding to LDLR [59]. In this study, the protein and mRNA abundance of ApoE and LDLR and the mRNA abundance of MTP and ApoB100 significantly increased in the dairy cows with fatty liver. Our results demonstrated lipid transport was affected after hepatic steatosis happened to dairy cows. It reports that dysregulation of lipid metabolic pathways results in the development of hepatic steatosis and contributes to the development of chronic hepatic inflammation, insulin resistance and liver damage [60]. The aforementioned studies showing that hepatic steatosis decreased SIRT1 activity suggest that the SIRT1-SREBP-1c/PGC-1α signaling pathway could influence oxidative stress. Nevertheless, whether lipid deposition affects the oxidative stress through the SIRT1-SREBP-1c/PGC-1α signaling pathway remains to be investigated in the future study.

Based on these results it can be concluded that SIRT1/PGC-1α/SREBP-1c and redox are involved in fatty liver dairy cows by promoting hepatic fatty acid synthesis, impairing fatty acid β-oxidation and reducing lipid transport. Limitations of the present study is the relatively small sample size. However, this study provides a foundation for future investigations of lipid metabolism disorder disease during the transition period.

## 5. Conclusions

These results demonstrate that decreased SIRT1 associated with hepatic steatosis promotes hepatic fatty acid synthesis, inhibits fatty acid β-oxidation and reduces lipid transport. Hence, SIRT1 may represent a novel therapeutic target for the treatment of fatty liver disease in dairy cows.

## Figures and Tables

**Table 1:** Clinical observations of control and mild fatty liver cows.

Groups	Milk Ketones	Feed Intake	Milk Production	Health Status	Reproductive Performance	Other Disease Conditions
Control	0	0	0	0	0	0
Mild fatty liver	+	−	−	−	−	0

The symbols + and − mean positive and negative association, respectively. The 0 means no association.

**Table 2:** The basic diet formulation, %.

Item	Content	
	Prenatal	Postpartum
Silage	31.4	40.0
Guinea grass	23.4	
Corn	19.6	35.0
Wheat bran	10.0	8.0
Soybean meal	2.0	5.0
Sunflower	11.5	8.0
NaCl	0.8	1.0
Premix1	1.3	1.8
NaHCO3		1.2
Total	100.00	100.00
	Energy (%) ^1^	
NEL(MJ/Kg) ^2^	5.7	6.7
Crude protein	11.3	15.2
Neutral detergent fiber	50.2	33.45
Acid detergent fiber	28.5	17.2
Ca	0.3	0.7
P	0.3	0.5
NFC Non fiber carbohydrate	28.0	40.4
RDP Neutral detergent fiber	7.0	7.4
NFC/RDP Nonfiber carbohydrate/ Neutral detergent fiber	4.0	5.5

^1^ The premix provided the following per kg of diets: VA 200,000 IU, VD 70,000 IU, VE 1000 IU, Fe 2000 mg, Cu 600 rng, Zn 2400 mg, Mn 1300 mg, I 6 mg, Se 17 mg, CO 7 mg; ^2^ NEL was a calculated value and others were measured values.

**Table 3:** Primers sequences qRT-PCR used in this experiment.

Genes ^1^	Primer Sequences (5′-3′)	Gene Bank Accession no.	Amplicon (bp)	Annealing Temperature (°C)
SIRT1	Forward: TATGGAGTGACATAGAGTGTGCT	XM_015461011.1	143	57
	Reverse: GTCGCTACACCACTTCAATCC			
SREBP-1c	Forward:CGACACCACCAGCATCAACCACG	NM_001113302.1	119	62
	Reverse: GCAGCCCATTCATCAGCCAGACC			
ACCα	Forward: TGCTGAATATCCTCACGGAGCT	NM_174224.2	212	60
	Reverse: CGACGTTTCGGACAAGATGAGT			
FAS	Forward: ACAGCCTCTTCCTGTTTGACG	NM_174662.2	226	59
	Reverse: CTCTGCACGATCAGCTCGAC			
ACSL	Forward: TCGGAACTGAAGCCATCACC	NM_001076085.1	173	63
	Reverse: GCCTCGTTCCAGCAGATCAC			
CPT-1	Forward: ACGCCGTGAAGTATAACCCT	NM_001304989.1	119	60
	Reverse: CCAAAAATCGCTTGTCCCTT			
CPT-2	Forward: TGAACATCCTCTCCATCTGG	NM_001045889.2	188	58
	Reverse: GGTCAACAGCAACTACTACG			
ACO	Forward: TACGGAGGGATGAGGAGTGT	NM_001205495.1	143	64
	Reverse: TCTCAGGAAGCGAGTTTGG			
LCAD	Forward: GGTCCACAGCACAGACTTGGT	NM_001076936.1	151	56
	Reverse: GGAATTGGCTAGGCTTGTGATC			
L-FABP	Forward: AAGTACCAAGTCCAGACCCAG	NM_175817.3	111	61
	Reverse: CACGATTTCCGACACCC			
LDLR	Forward: GCTGTTCTGCCTTTCTCCTT	NM_001166530.1	228	65
	Reverse: ACTTTCTCCCCTGACCCTTG			
Apo-100	Reverse: GATACTCAGAACGGAGCAAT	XM_019969506.1	222	58
	Forward: GCACCAATCAGATAACAGGA			
ApoE	Reverse: TCCTGAATGACCTGGGTGTTG	XM_005219148.3	219	62
	Forward: TCTGTGGGTTGCCGTGGTG			
MTTP	Reverse: CAGTTTGCAGCCTTGGTTCTG	NM_001101834.1	201	56
	Forward: TTCAAAAGCACCGAGAGCGTT			
PGC-1α	Reverse: GACCACAAATGATGACCCTC	NM_177945.3	123	60
	Forward: GGTTTGGCTTGTAGATGTT			
Nrf1	Reverse: TTTTAGTAACCCTGATGGC	NM_001098002.2	198	57
	Forward: GCTTGCGTTGTCTGGATG			
TFAM	Reverse: TGGCACATCACAGGTAAA	NM_001034016.2	137	63
	Forward: GTTCCTCCCAAGATTTCA			
MnSOD	Reverse: TTCAATAAGGAGCAGGGAC	NM_201527.2	234	64
	Forward: CAGTGTAAGGCTGACGGTTT			
Cu/Zn SOD	Reverse: GAAGAGAGGCATGTTGGAGA	NM_174615.2	221	60
	Forward: CCAATTACACCACGAGCCAA			
CAT	Reverse: AGATACTCCAAGGCGAAGGTG	NM_001035386.2	120	61
	Forward: AAAGCCACGAGGGTCACGAAC			
GSH-Px	Reverse: GCGGGAGCAGGACTTCTACGA	NM_001101113.2	137	65
	Forward: CCCGATAGTGCTGGTCTGTGAA			
PPARα	Reverse: GGGTTTTCTTAGGCTTTT	NM_001034036	176	60
	Forward: AGTCCATCCCTGGGTTTG			
RXRα	Reverse: GGCAGATGTTGGTGACGGG	NM_001304343	163	62
	Forward: GGCGAGAGCGAGGTGGAGT			
β-actin	Reverse: GCCCTGAGGCTCTCTTCCA	NM_173979.3	101	59
	Forward:GCGGATGTCGACGTCACA			

^1^ SIRT1 = sirtuin 1; SREBP-1c = sterol regulatory element binding transcription factor 1; ACCα = acetyl-CoA carboxylase alpha; FAS = fatty acid synthetase; ACSL = acyl-CoA synthetase long chain family member; CPT1 = carnitine palmitoyltransferase 1; CPT2 = carnitine palmitoyltransferase 2; ACO = acyl coenzyme A oxidase; LCAD = long chain acyl-CoA dehydrogenase; L-FABP = liver fatty acid binding proteins; LDLR = low density lipoprotein receptor; ApoB100 = apolipoprotein B 100; ApoE = apolipoprotein E; MTTP = microsomal triglyceride transfer protein; PGC-1α = peroxisome proliferator-activated receptor γ coactivator-1 alpha; Nrf1 = nuclear respiratory factor 1; TFAM = transcription factor A, mitochondrial; MnSOD = superoxide dismutase 2, mitochondrial; Cu/Zn SOD = superoxide dismutase 1; CAT = catalase; GSH-Px = glutathione peroxidase 7; PPARα = peroxisome proliferator-activated receptor alpha; RXRα = retinoid X receptor alpha.

**Table 4:** Milk production and milk component of control and fatty liver dairy cows.

Parameter	Control	Fatty Liver	*p*-Value
DMI, kg/d	23.17 ± 3.24	18.45 ± 2.84	<0.01
Milk production, kg/d	28 ± 7	20 ± 6	<0.01
Milk component			
Fat, %	3.48 ± 0.35	3.33 ± 0.32	0.39
Protein, %	3.25 ± 0.21	2.91 ± 0.34	<0.01
Lactose, %	4.96 ± 0.11	4.88 ± 0.13	0.91
MUN ^1^ (mg/100 mL)	13.76 ± 1.91	11.52 ± 1.43	0.01
SCC (×10^3^/mL)	112.35 ± 20.23	365.61 ± 43.25	<0.01

^1^ MUN, milk urea nitrogen; SCC, somatic cell count.

**Table 5:** Genes mRNA abundance for antioxidation activity of control and fatty liver cows (means ± SEM).

Genes	Control (n = 6)	Fatty Liver (n = 6)
Cu/Zn SOD	1.00 ± 0.12	0.31 ± 0.09
CAT	1.00 ± 0.06	0.21 ± 0.15 **
GSH-Px	1.00 ± 0.20	0.09 ± 0.06 **
Mn SOD	1.00 ± 0.13	0.06 ± 0.03 **

Note: ** represent *p* < 0.01.
